# Optimizing passive acoustic sampling of bats in forests

**DOI:** 10.1002/ece3.1296

**Published:** 2014-12-02

**Authors:** Jérémy S P Froidevaux, Florian Zellweger, Kurt Bollmann, Martin K Obrist

**Affiliations:** 1 WSL Swiss Federal Institute for Forest, Snow and Landscape Research, Biodiversity and Conservation BiologyZürcherstrasse 111, CH-8903, Birmensdorf, Switzerland; 2 University of Montpellier II2 Place Eugène Bataillon, Cedex 05, F-34095 Montpellier, France; 3 Forest Ecology, Institute of Terrestrial Ecosystems, Department of Environmental Systems Science, ETH ZürichCH-8092, Zürich, Switzerland

**Keywords:** Activity, cost-effectiveness, echolocation, forest microhabitats, inventory, species richness

## Abstract

Passive acoustic methods are increasingly used in biodiversity research and monitoring programs because they are cost-effective and permit the collection of large datasets. However, the accuracy of the results depends on the bioacoustic characteristics of the focal taxa and their habitat use. In particular, this applies to bats which exhibit distinct activity patterns in three-dimensionally structured habitats such as forests. We assessed the performance of 21 acoustic sampling schemes with three temporal sampling patterns and seven sampling designs. Acoustic sampling was performed in 32 forest plots, each containing three microhabitats: forest ground, canopy, and forest gap. We compared bat activity, species richness, and sampling effort using species accumulation curves fitted with the clench equation. In addition, we estimated the sampling costs to undertake the best sampling schemes. We recorded a total of 145,433 echolocation call sequences of 16 bat species. Our results indicated that to generate the best outcome, it was necessary to sample all three microhabitats of a given forest location simultaneously throughout the entire night. Sampling only the forest gaps and the forest ground simultaneously was the second best choice and proved to be a viable alternative when the number of available detectors is limited. When assessing bat species richness at the 1-km^2^ scale, the implementation of these sampling schemes at three to four forest locations yielded highest labor cost-benefit ratios but increasing equipment costs. Our study illustrates that multiple passive acoustic sampling schemes require testing based on the target taxa and habitat complexity and should be performed with reference to cost-benefit ratios. Choosing a standardized and replicated sampling scheme is particularly important to optimize the level of precision in inventories, especially when rare or elusive species are expected.

## Introduction

Species richness is a widely used variable in ecological research (Purvis and Hector 2000) and a key indicator of biological diversity in monitoring programs (Yoccoz et al. 2001). However, a species count often underestimates the true number of species present (Kery and Schmid 2006), in particular, in rare, elusive, and nocturnal taxa.

In the past three decades, acoustic survey methods have become increasingly popular in faunistic biodiversity studies. Today, a wide range of terrestrial animals that produce sounds may be acoustically sampled, most prominently bats (Obrist et al. 2004), insects (Chesmore and Ohya 2004), amphibians (Huang et al. 2009). and birds (Wimmer et al. 2013). Apart from being noninvasive and cost-effective, acoustic sampling is superior to other methods, such as capturing, which is difficult to implement in cluttered habitats as forests and tends to underestimate species richness (MacSwiney et al. 2008). With passive acoustic sampling techniques (researcher absent), considerable quantities of data about species presence, abundance, and species behavior at large spatiotemporal scale can be collected. Thus, acoustic methods can be used to estimate population density (Marques et al. 2013), study animal behavior (Lynch et al. 2013), or assess and track changes in species composition in a context of habitat modification and climate change (Blumstein et al. 2011).

Recent technological advancements have allowed acoustic studies of bats to be more effective and accurate. Employing new technologies, several new bat detectors have emerged which are increasingly sensitive and include omnidirectional microphones, thus improving overall bat detection (Britzke et al. 2013). In parallel with improvement in ultrasonic detectors, different types of software for bat species identification based on specific algorithms have been developed to deal with the interspecific convergence in bat call design and intraspecific structural variation (Vaughan et al. 1997; Russo and Jones 1999; Parsons and Jones 2000; Obrist et al. 2004; Preatoni et al. 2005). While some applications help ecologists to analyze bat echolocation calls with automated extraction of call features, others allow an automatic classification and identification of bat echolocation call recordings by statistically associating unknown calls with reference calls. Despite constantly improving methods for bat detection and species identification, extrinsic factors may still bias the acoustic sampling. These factors include habitat features such as cluttered forests which induce bats to change the echolocation call structure and reduce call intensity (Brigham et al. 1997; Schnitzler and Kalko 2001), thereby reducing detection and making species identification more difficult.

To enhance acoustic sampling in forests, effects of position, orientation, and number of detectors on bat detection must be accounted for. While Weller and Zabel (2002) highlighted the importance of deploying detectors well above ground level and orienting microphones toward cluttered space, Duchamp et al. (2006) showed the need to laterally deploy at least two detectors in heterogeneous forest stands. The vertical stratification of bat activity in forests has been demonstrated for communities in North America (Kalcounis et al. 1999), Australia (Adams et al. 2009), and Europe (Müller et al. 2013). This means that sampling forests require detectors at both the ground layer and higher strata up to the canopy (Britzke et al. 2013). Despite evidence that bats use three-dimensions of forest space to forage (Jung et al. 2012), no acoustic sampling scheme has been specifically evaluated for the ability to inventory forest bats. Given that forests provide both roosting and foraging habitats for a majority of bat species (Dietz et al. 2007), there is strong incentive for scientists, wildlife managers, and agencies to know the type of acoustic sampling methods that best suit project-specific goals regarding forest bat inventories.

The aims of our study were (1) to compare activity patterns of bat guilds among forest microhabitats; (2) to evaluate the utility of different acoustic sampling schemes (i.e., number of bat species detected) and effectiveness (i.e., number of nights invested) by testing different sampling designs (i.e., spatial positions of the bat detectors) associated with various temporal sampling patterns (i.e., time windows to sample during a night); and (3) to assess the time and cost of assessing bat species richness in forests by implementing the best sampling schemes at different forest locations. Forest microhabitat preferences of bats depend on foraging strategy and ecomorphological traits (Norberg and Rayner 1987). Consequently, we hypothesized to detect more species when employing a sampling protocol that takes vertical and horizontal stratification of forest habitats into account. Furthermore, as bat activity varies temporally during the night and among species (Hayes 1997; Skalak et al. 2012), we hypothesized to detect more species by extending the sampling pattern from 4 h to full-night recordings. Finally, we hypothesized to find a trade-off between material and labor costs depending on the spatiotemporal requirements of the respective sampling schemes.

## Material and Methods

### Study area and site selection

The study was conducted in the Canton of Aargau (47°14′–47°62′N, 7°71′–8°46′E; 1404 km^2^), in northwestern Switzerland. The area lies in the biogeographical regions of the Swiss lowlands and Jura Mountains, with altitudes ranging from 260 to 910 m a.s.l. More than one-third of the area (37%) is covered by mixed deciduous and coniferous forests, of which 80% are managed for wood production and the remaining 20% for other purposes, such as biodiversity conservation or recreation. The most abundant tree species are *Fagus sylvatica* (32%), *Picea abies* (26%), and *Abies alba* (14%) (Departement Bau, Verkehr und Umwelt 2010).

**Figure 1:**
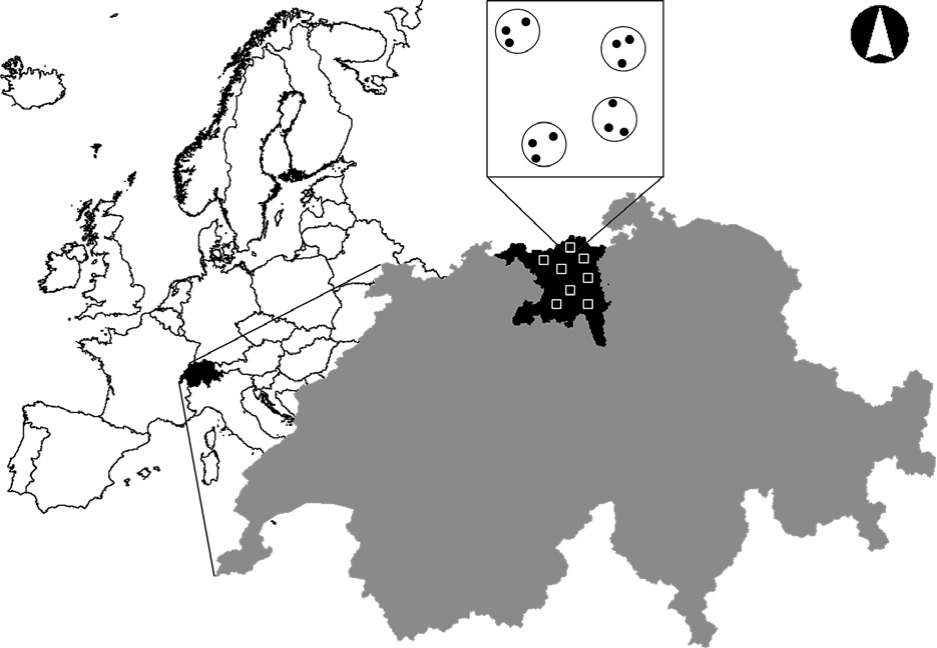
Stratified random sampling design: sampling sites (•), within plots (○) in a sample of km^2^ cells (□) located in the Canton of Aargau, northwestern Switzerland (not to scale).

We implemented a stratified random sampling design (Fig. 1) to select eight cells of 1 km^2^ (mean distance between cells: 13.6 km), based on the national mapping grid. We considered only cells with at least 50% forest cover, as delineated by the digital mapping product VECTOR25 (Swisstopo 2013), and randomly selected cells with respect to the altitudinal gradient. To avoid potential biases arising from foraging areas other than forests, candidate cells were at least 100 m from any bodies of water. In each cell, we randomly selected four plots that were entirely located within forests. In each plot, we identified three sites each representing a particular microhabitat: two were located in the forest interior: (1) the forest ground and (2) the respective canopy above; and (3) one in the center of a nearby forest gap.

To minimize confounding edge effects, we selected sites with minimal distances of 50 m and 20 m from forest edges and forest roads, respectively. Within the 1-km^2^ cells, plots were 145–800 m (mean 429 m) apart, and within plots, forest and gap sites were located 46–140 m (mean 81 m) from each other. Only deciduous and mixed forests were considered. The area of forest gaps ranged from 450 to 3950 m^2^ (mean 1318 m^2^).

### Acoustic sampling

To record bat echolocation calls, we used 12 ultrasound detectors (Batlogger; Elekon AG, Lucerne, Switzerland). Detectors contained water-resistant and omnidirectional microphones sensitive from 10 to 150 kHz (±5 dB). Each detector was placed in a Strongbox (Elekon AG, Lucerne, Switzerland), which provided protection from weather, and augmented energy autonomy to about 135 h using three Li-ion cells.

Bat activity was recorded on 71 full nights between 4 June and 29 August 2013. No sampling was undertaken on nights with rainfall and minimum temperatures below 7°C. Detectors were programmed to record sounds automatically between 21:30 and 05:30, triggered by tonal ultrasound signals. Using 12 ultrasound detectors, 1 km^2^ could be sampled per night (Fig. 1). We sampled each square km for three or four consecutive nights before moving to the next square km. The detectors were randomly switched between sites after each rotation to avoid possible bias. Each cell was sampled 6–12 nights during the field session.

In the forest ground and the forest gap, detectors were mounted on a pole 1.35 m above the ground. For the canopy sites, we selected deciduous trees representative of the forest stand and used a slingshot, ropes, and a pulley system to install the detectors in the upper canopy. The height of the detectors ranged from 13.5 to 30 m (mean 18.9 m), which corresponds to 85% of the mean stand (30 × 30 m) height, as calculated from a digital surface model (C. Ginzler, pers. comm.).

### Echolocation analysis

To identify bat echolocation calls to the species, we used the software BatScope (Boesch and Obrist 2013). BatScope cuts every recorded series of echolocation calls (here after simply termed “sequence”) into single calls and processes them into spectrograms (0.31 kHz × 0.16 ms resolution), from which it extracts 23 numeric variables. Based on three different classification algorithms – *support vector machine (SVM)*, *K nearest neighbor (KNN)*, and *quadratic discriminant analysis (QDA)* – calls are then classified to species, taking into consideration the respective variable values from 19,636 reference calls from 28 European species (Obrist et al. 2004). The correct classification rate of calls reaches 95.7% when only considering calls classified to the same species by all three classifiers. By doing so, 23.6% of all calls are being rejected from classification as being too ambiguous to identify (see Table 1 in Boesch and Obrist 2013).

**Table 1:** Number of sequences per bat species recorded in each microhabitat

		Forest gap		Forest ground		Canopy		Total	
					
Species	Guild	No. of sequences	%	No. of sequences	%	No. of sequences	%	No. of sequences	%
*Eptesicus spec*.	LRE	241	0.46	22	0.05	26	0.07	289	0.22
*Hypsugo savii*	MRE	7	0.01	0	0.00	3	0.01	10	0.01
*Myotis brandtii*	SRE	33	0.06	33	0.08	29	0.08	95	0.07
*Myotis bechsteinii*	SRE	1	0.00	2	0.00	3	0.01	6	0.00
*Myotis daubentonii*	SRE	211	0.40	570	1.36	280	0.79	1061	0.82
*Myotis emarginatus*	SRE	47	0.09	228	0.54	137	0.38	412	0.32
*Myotis myotis*	SRE	221	0.42	578	1.38	24	0.07	823	0.63
*Myotis mystacinus*	SRE	19	0.04	132	0.32	19	0.05	170	0.13
*Myotis nattereri*	SRE	4	0.01	13	0.03	0	0.00	17	0.01
*Nyctalus spec*.	LRE	223	0.42	13	0.03	13	0.04	249	0.19
*Pipistrellus kuhlii*	MRE	1420	2.70	436	1.04	413	1.16	2269	1.75
*Pipistrellus nathusii*	MRE	8954	17.04	1271	3.04	773	2.17	10,998	8.46
*Pipistrellus pipistrellus*	MRE	41,111	78.24	38,402	91.76	33,827	94.94	113,340	87.17
*Pipistrellus pygmaeus*	MRE	38	0.07	130	0.31	75	0.21	243	0.19
*Plecotus spec*.	SRE	13	0.02	20	0.05	8	0.02	41	0.03
*Vespertilio murinus*	LRE	4	0.01	0	0.00	0	0.00	4	0.00
Total		52,547		41,850		35,630		130,027	

LRE, long-range echolocators; MRE, middle-range echolocators; SRE, short-range echolocators.

After automatic classification of calls, we performed a semiautomatic identification of bat sequences with different filter combinations (e.g., the sequence contains >10 calls, and >80% of the calls are classified with >70% confidence to a single species) to associate sequences to the best taxonomic level possible: species, species groups, genus, and genus groups.

A manual control with BatScope and RavenPro (Charif et al. 2004) was used (1) at the call level to avoid misclassification of background noises to bat echolocation calls and to bring in expert knowledge to distinguish obvious calls (e.g., social calls); (2) at the sequence level to test each filter for errors in the semiautomatic verification process. To that end, we manually verified 10% of the sequences that were automatically classified to species with easy discernibility (e.g., *Pipistrellus pygmaeus*, *Pipistrellus pipistrellus*, *Myotis myotis*) and 33% of the sequences from species that are easily confused with others (e.g., *Pipistrellus nathusii, Pipistrellus kuhlii* or *Myotis brandtii, Myotis mystacinus*, etc.).

As echolocation calls are similar between some species (Obrist et al. 2004), we grouped (1) *Plecotus auritus* and *Plecotus austriacus* into *Plecotus* sp.; (2) *Nyctalus noctula* and *Nyctalus leisleri* into *Nyctalus* sp.; and (3) *Eptesicus serotinus* and *Eptesicus nilssoni* into *Eptesicus* sp. We further classified bats into three different guilds, according to their clutter resistance and echolocation range (Schnitzler and Kalko 2001): short-range echolocators (SRE), middle-range echolocators (MRE), and long-range echolocators (LRE) (for details, see Frey-Ehrenbold et al. 2013).

### Statistical analyses

All statistical analyses were undertaken using R 2.15 (R Development Core Team 2013). We assessed the activity of the three guilds by taking into account sequences from all taxonomic levels assignable to a guild. As a single bat may forage around a microphone for extended periods, we quantified activity by counting the number of 5-min intervals containing sequences of a given species per night. Thus, the maximum activity per night for a given species was 96 (8 h × 60 min/5 min). As data on activity were not normally distributed even after transformations but followed a Poisson distribution, we used generalized linear mixed models (GLMMs) (function *glmer*, R package *lme4*) with a Poisson distribution to analyze the differences between the guilds’ activities as a function of microhabitat. Ambient temperature and type of microhabitat were considered as fixed effects, whereas the number of sites (32 per microhabitat) and nights (6 to 12) were implemented as random effects to avoid pseudo-replications. We applied a stepwise regression method to select the best models using the Akaike information criterion (Burnham and Anderson 2004), choosing the model with the fewest parameters when models were considered equivalent (*ΔAIC* < 2).

We estimated bat species richness and evaluated the minimum sampling effort required to achieve a complete inventory of bats using species accumulation curves (Moreno and Halffter 2000; Gotelli and Colwell 2001). For species grouped together in the same genus (i.e., *Plecotus* sp., *Nyctalus* sp., and *Eptesicus* sp.), a group was considered to be one taxon in the analysis. We used the function *specaccum* in the R package *vegan* to calculate curves for single microhabitats and for combinations of microhabitats belonging to the same plot, thereby evaluating three different temporal sampling patterns: (1) recording over the full night, (2) recording for only the first 4 h after sunset (21:30–01:30), and (3) recording for 4 h split into two sessions, the first after sunset (21:30–23:30) and the second before sunrise (03:30–05:30). The number of sampling nights was considered as the sampling effort. Sampling order was randomized 1000 times to avoid possible order specific bias and to produce smooth rarefaction curves. To account for potentially unequal numbers of sampling repetitions (e.g., due to low temperature) and at the same time allow for extrapolating and comparing species accumulation curves, we fitted the Clench equation (Soberon and Llorente 1993) to our data:


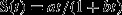
(1)

where S(*t*) is the predicted number of species at sampling effort *t*, *a* is the rate of increase at the beginning of sampling, and *b* is a parameter related to the shape of the accumulation of new species during the sampling. The Clench equation is appropriate to use when the probability of adding new species decreases with the number of species already detected, but increases over time (Soberon and Llorente 1993). Model parameters *a* and *b* were obtained by fitting nonlinear least square procedures (function *nls*) using the R package *stats*. Finally, we fitted species accumulation curves for distinct microhabitats and for combinations of microhabitats for the three temporal sampling patterns, by averaging the corresponding model's parameters. Because reaching 100% species richness in forests requires a large sampling effort (Moreno and Halffter 2000), the sampling effort was considered satisfactory when 90% of the estimated species richness (asymptote of the curve), occurring either at the site level or at the plot level, was reached (Skalak et al. 2012).

To establish the number of sampling plots required for a complete species inventory at the 1-km^2^ scale, we performed the same procedure using data resulting from the best sampling schemes to calculate the species richness occurring at the plot level. We built species accumulation curves for different numbers of sampling plots and considered the number of nights as the sampling effort.

### Sampling costs estimation

Considering only the number of sampling nights as effort when using passive acoustic methods drastically underestimates the total effort invested. We thus compared the time and labor cost required for implementing the best sampling schemes in different sampling plots to assess bat species richness at the 1-km^2^ scale. We added equipment costs to determine total costs. We associated the number of nights (N_*n*_) and the number of plots (N_*p*_) required in the sampling schemes previously evaluated to be optimal to (1) the time related to the fieldwork (T_*f*_) (see details in Table S1, Supporting Information) and (2) the time required for the echolocation analysis (T_*a*_). To estimate T_*a*_, we calculated the mean number of sequences recorded per night and per plot (X_*s*_) according to the sampling scheme used. Then, we estimated the average time required to identify a certain number of bat sequences to the species level, by quantifying the mean number of workdays required for three users of BatScope. Here, we took into account the time needed for the manual species identification and for developing filters, which allowed for automatic identification. The results were converted into time/sequence (A). Finally, we measured the total time invested (T_*t*_):


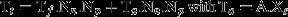
(2)

Given an average salary for skilled technicians of 50 € per hour and for scientific experts of 100 € per hour, we estimated the labor costs of forest bat inventories, and the total cost, by adding the cost of detectors (Batlogger: 1645 €/unit).

## Results

Using 12 detectors during 71 nights at 96 sampling sites, we recorded 145,433 bat sequences containing a total of 2,064,188 bat calls. We identified 129,671 sequences (89.2%) to the species level, 10,948 sequences (7.5%) to a species group, 4128 sequences (2.8%) to the genus level, and 168 sequences (0.1%) to a genus group. A total of 518 sequences contained only one bat call, or calls that were unidentifiable. Thus, we assigned them to the order Chiroptera (0.4%).

In total, 16 of 20 bat species recorded for the Canton of Aargau were detected in our study (Table 1). Among these 16 bat species, three belonged to the LRE guild, five to the MRE guild, and eight to the SRE guild. The most frequent species identified belonged to the genus *Pipistrellus*, with 87.2% of all sequences assigned to *Pipistrellus pipistrellus*, 8.5% to *Pipistrellus nathusii*, and 1.8% to *Pipistrellus kuhlii*. As *Pipistrellus pipistrellus* was present in all sites and dominant in each microhabitat, we excluded this species from the analyses of activity to have a better understanding of the other species belonging to the MRE guild.

### Bat activity

**Figure 2:**
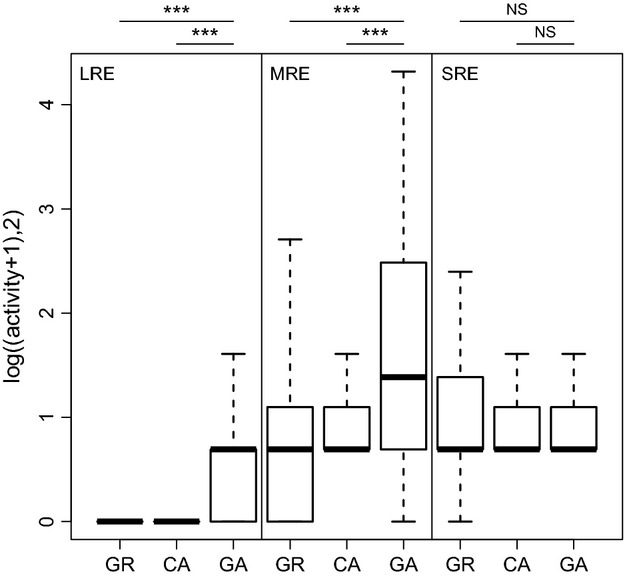
Activity measures of different bat guilds (LRE: long-range echolocators, MRE: middle-range echolocators without *P. pipistrellus*, SRE: short-range echolocators) for the three microhabitats (GR: forest ground, CA: canopy, and GA: forest gap). ***p < 0.001. NS, not significant.

The activity of the LRE and the MRE guilds was best explained by models that included the effect of the microhabitat (Table 2). While the activity of the SRE guild did not differ among microhabitats, the LRE and MRE guilds showed a preference for forest gaps (Fig. 2). Within guilds, activity did not differ between the forest ground and the canopy.

**Table 2:** Predictors of GLMMs with AIC explaining differences in activity per guild

Models	No. of parameters	LRE		MRE		SRE	
		
		*AIC*	*ΔAIC*	*AIC*	*ΔAIC*	*AIC*	*ΔAIC*
Temperature + microhabitat	6	699.5	0.0	8302.4	0.3	5259.4	0.1
Microhabitat	5	**701.2**	1.7	**8302.0**	0.0	5267.7	8.4
Temperature	4	749.0	49.5	8344.9	42.8	**5259.0**	**0.0**

Guilds: LRE, long-range echolocators; MRE, middle-range echolocators; SRE, short-range echolocators. Bold numbers indicates best fitting models for each guild.

### Species richness

**Figure 3:**
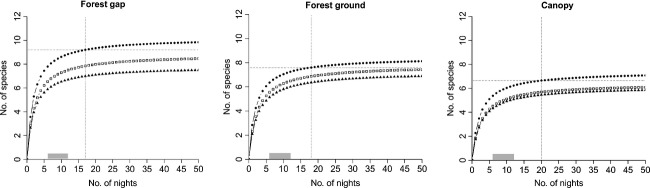
Averaged bat species accumulation curves by microhabitat (n = 32) for the full-night sampling (•), the first 4 h sampling (□), and double 2-h sampling after sunset and before sunrise (▴). Horizontal doted lines represent the species richness threshold of 90% (see Material and Methods), and vertical dotted lines, the corresponding number of nights required (sampling effort). Grey bars correspond to the numbers of nights invested in the field. Parameters of the curves are described in the Table S2, Supporting Information.

**Figure 4:**
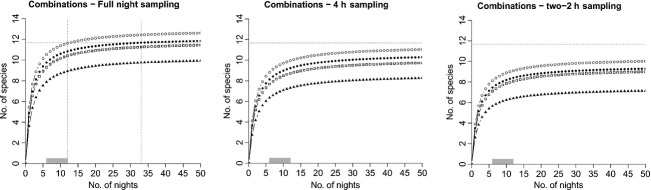
Averaged bat species accumulation curves for different temporal sampling patterns as function of sampling nights in different combinations of two microhabitats: “Forest gap + Forest ground” (•), “Forest gap + Canopy” (□), and “Forest ground + Canopy” (▴). Open circles (○) represent the combination of all three microhabitats. Horizontal doted lines represent the threshold of 90% (see Material and Methods), and vertical dotted lines, the corresponding number of nights (sampling effort) when sampling in four (left line) or three plots (right line), respectively. Grey bars correspond to the range of number of nights invested in the field. Parameters of the curves are described in the Table S3, Supporting Information.

Site-specific estimates of bat species richness proved highest in gaps, followed by forest ground, and canopy (Fig. 3; Appendix S1, Supporting Information). At the plot level, we found bat species richness to be highest when all three sites were combined (Fig. 4). However, when considering only two sites, the best site combination for assessing bat species richness within a plot was the “Forest gap + Forest ground” combination, in which we recorded 90% of the full species assemblage, albeit with considerable effort of 33 sampling nights.

Bat species richness increased when the sampling duration was extended, regardless of the microhabitats or their combination (Figs. 3 and 4). Full-night sampling was necessary to make a good estimate of the number of species present. Further, by comparing 4-h sampling (first half of the night) to the double 2-h sampling (2 h after dusk and 2 h before dawn), we found that species richness was on average higher during the first part of the night (Fig. 3).

### Sampling effort

The minimum sampling effort required to record 90% of the species occurring at a site varied between the microhabitats and the temporal sampling patterns (Fig. 3). We found that the sampling effort needed was lowest in the forest gap (17 nights), followed by the forest ground (18 nights), and the canopy (20 nights). Only with full-night sampling was it possible to reach 90% of the total species richness.

To reach 90% of the total bat species richness at the plot scale, only two sampling schemes proved to be adequate: full-night sampling either in the three microhabitats (12 nights) or in the “Forest gap + Forest ground” combination (33 nights) (Fig. 4). With the other schemes, 90% of the total bat species richness could not be achieved or required an effort of more than 50 nights.

### Sampling costs

**Figure 5:**
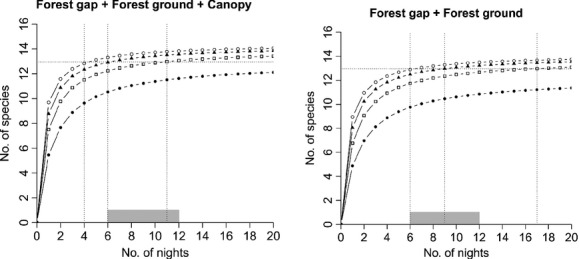
Averaged bat species accumulation curves for the best sampling schemes as function of sampling nights for different numbers of sampling plots to implement in a 1-km^2^ cell: one plot sampled (•), two plots sampled (□), three plots sampled (▴), and four plots sampled (○). Horizontal doted lines represent the threshold of 90% (see Material and Methods) of the averaged total species richness occurring in a 1-km^2^ cell, and vertical dotted lines, the corresponding number of nights (i.e., the required sampling effort). Grey bars correspond to the number of nights invested in the field. Parameters of the curves are described in the Table S4, Supporting Information.

Based on the ideal sampling scheme at the plot scale, we estimated the number of plots required to assess bat species richness at the square km scale and the corresponding temporal and financial resources needed. We found two trends (Table 3). First, when increasing the number of plots, the time for field management and analysis decreased due to a lower number of sampling nights required (Fig. 5). Second, considering material costs, the costs turn out to be very similar between the different scenarios. As sampling a single plot would never lead to reaching the required total species richness, the costs for this scenario were not calculated. By considering together the labor costs with the detector costs, we found that about 37 000 € (±1465 €) are needed when sampling in the three microhabitats regardless of the number of plots and 34 000 € (±633 €) when considering only the “Forest gap + Forest ground” combination.

**Table 3:** Time investment and costs for different sampling schemes to reach 90% of the estimated bat species richness occurring at the km^2^ scale in a forest landscape

Sampling scheme	No. of plots	No. of nights required	Field management (h)	Time for analysis (h)	Labor cost (€)	Detector cost (€)	Total cost (€)
GA + GR + CA Full night [2069]	1	–	–	–	–	–	–
	2	11	14.8	252.9	26,030	9870	35,900
	3	6	9.6	206.9	21,170	14,805	35,975
	4	4	6.9	183.9	18,735	19,740	38,475
GA + GR Full night [1524]	1	–	–	–	–	–	–
	2	16	15.2	270.9	27,850	6580	34,430
	3	9	8.7	228.6	23,295	9870	33,165
	4	6	7.2	203.2	20,680	13,160	33,840

GA, Forest gap; GR, Forest ground, CA, Canopy.

The mean number of bat sequences recorded per night and per plot is given in square brackets. Field management included different field aspects (Table S1, Supporting Information) and was based on the assumption that each field session lasts two nights. Costs are based on salaries of €50/h for fieldwork and €100/h for acoustic analyses of the respectively recorded sequences.

## Discussion

We found that using passive acoustic methods to accurately register species presence depends on the temporal and spatial replication of a standardized sampling technique. Studies that restrict their surveys to a particular time window during the night or a particular forest layer or microhabitat (e.g., forest gap) will likely underestimate true species richness. The observed species richness deviates from the true species richness *N* due to each species’ detection probability *p*, which varies among the species present (MacKenzie and Kendall 2002). With temporal and spatial replication, we strived to compensate for detection probabilities that differ between species. It is well known that detection probabilities of bats are less than one: deviations usually stem from observer biases, weather conditions, species characteristics, and abundances, and habitat variability (Meyer et al. 2011). Observer biases can be excluded in our study because we used an automated technique (MacSwiney et al. 2008). The influence of cold and rainy weather on the activity of bats was controlled using only data from dry nights with minimum temperature at or above 7°C. Habitat variability was accounted for by the sampling design within a plot and the replication within a square km. Thus, we assume that the pronounced differences in species abundances we calculated, rather than their detectability, have had the dominant effect on detecting species. We believe that our observed species richness value is a good proxy for the true resident species richness (excluding sporadic migrants), as we were able to record 16 species on only 1.5% of the total forested study area in the canton of Aargau, for which, in total, 20 species have been documented over the last decades.

### Where to sample bats in forests? – Selection of sampling design

Our results provide ample evidence that the three-dimensional structure of forests must be sampled to adequately record bat communities. These findings corroborate Jung et al. (2012), which show that bat species exhibit microhabitat preferences depending on their echolocation type and wing morphology (Norberg and Rayner 1987). As we showed with the activity measurement of bat guilds, forest gaps constitute an important microhabitat for the majority of bat species foraging on aerial insects: bats with high flight speed, low maneuverability (e.g., *Nyctalus* sp.) known to forage in open space or in open forests, used forest gaps like some species known to be edge specialists (e.g., *Pipistrellus* sp.) (Jung et al. 2012; Mehr et al. 2012). On the forest ground, however, bat species with low flight speed and high maneuverability prevail (e.g., *Myotis* sp.) (Mehr et al. 2012; Müller et al. 2013), of which most are gleaning foragers (Schnitzler and Kalko 2001). Finally, both edge specialists and forest specialists forage in the canopy (Müller et al. 2013). For bats, the canopy provides foraging opportunities along a horizontal edge shaped by the roughness of the canopy (Jung et al. 2012).

Although sampling in all three microhabitats produced the best results, surprisingly, sampling a combination of only two microhabitats, the forest gap and the forest ground, produced results similar to the ideal design, enabling the recording of 90% of the total species richness with a marginal increase in effort. As expected, there was a high complementarity of species recorded in the forest gap and those either in the forest ground or in the canopy. However, in contrast to our expectations, we found a high similarity between the species detected in the canopy and those in the forest ground. This cannot be explained by a possible bias of the detection because, even though we installed the detectors in the upper part of the canopy and not above, the high sensitivity of the detectors (Adams et al. 2012) allowed us to detect both species foraging within the canopy and species foraging on the ground (e.g., forest specialist), as well as those foraging above it (e.g., edge specialists and open space foragers) (Adams et al. 2009; Müller et al. 2013). The main reason for this could arise from our canopy settings: the neighboring density of vegetation was sometimes relatively high, reducing maneuverability, and thus, the presence of bats in the canopy.

### When to sample bats in forests? – Choice of temporal sampling pattern

Acoustic sampling of bats is commonly conducted in either of three different ways: (1) at dusk only; (2) at dusk and dawn with a break in between; or (3) for the entire night. When sampling is performed during parts of the night, the recording time window usually targets the period with peak bat activity, thus assuming to record the majority of species. However, the temporal variation of bat activity is habitat and species-specific (Hayes 1997) as is the time of the emergence of bats (Jones and Rydell 1994). Skalak et al. (2012) recently showed that sampling the full night was essential to cover the bimodal peaks of bat activity, but also to record rare species having low detection probabilities. Our results fully support these findings. We demonstrated that independent of microhabitat, sampling for the entire night resulted in the maximum number of bat species recorded. This means that bat species richness is underestimated by sampling for only 4 h per night, even when taking into account the general bimodal peaks of bat activity.

### Cost-benefit considerations

Efforts to track changes in biodiversity are subject to the trade-off between the effort invested and the gain of information (Duelli and Obrist 1998). Despite the fact that sampling costs are a crucial argument for optimizing spatiotemporal samplings, few studies have monetarily valued the type of sampling used (Gardner et al. 2008).

Species richness estimates increase with sampling effort up to reaching an asymptotic level representing true total species richness present. In our study, increasing the number of plots from three to four in a forest inventory at the 1-km^2^ scale only marginally increased the estimated total species richness (Fig. 5; Table S4), which leads us to the conclusion that we have sampled the complete community. However, increasing the spatial replication to four forest locations using the best sampling scheme allowed us to reduce the number of sampling nights and thus to save time and money to estimate bat species richness. This likely reflects higher spatial than temporal variance of bat activity among events. Nevertheless, by adding the costs of devices required, the total costs tend to be balanced. There is a trade-off between the number of plots to sample for decreasing the time and consecutive costs spent in the field and for the analysis, and the number of detectors to implement, known to be expensive.

By explicitly accounting for the time and the costs of each step induced by the sampling effort required, we demonstrate that the processing time required to identify bat sequences remains the major constraint of the passive acoustic method. As for birds (Wimmer et al. 2013), species identification is challenging and time-consuming, despite the recent emergence of software for automated classification. To date, no software is robust enough to identify at the species level all bat sequences collected in the field. In this study, 10.8% of the total data recorded (i.e., 15,762 sequences) were not identified to species, leading to a slight underestimation of species richness (e.g., the two *Eptesicus* species present in Switzerland were grouped to only one taxa; numerous *Myotis* bat sequences were identified as species complexes). Efforts to improve the performance and availability of such software would substantially alleviate inventory costs and could leverage automated acoustic bat recording into the realm of broad monitoring programs. Finally, we also highlight the substantial effort required to assess bat species richness in forest habitat, even when deploying a large number of detectors. Due to the scarcity and/or the low detectability of some gleaning species (Meyer et al. 2011), a complete inventory is very demanding.

### Conclusion and recommendations

Adopting an effective sampling protocol to assess true species richness in a complex environment is a challenge, but at the same time it is a prerequisite for monitoring trends of biodiversity across space and time (Yoccoz et al. 2001). Our findings constitute an important step toward successfully implementing protocols that provide accurate inventories of bats. This is important given that they are known to be valuable bioindicators in times of global change (Jones et al. 2009). We propose the following recommendations to optimize acoustic sampling of bats in forests: (1) sample over the full night to achieve the most accurate estimate of species richness; (2) sample repeatedly in different forest microhabitats (forest gap, ground and canopy) reflecting the 3-D forest space used by bats; and (3) sample different forest locations. Taken together, this will allow us to determine species richness with less effort and at lower cost. Our approach is applicable to other fields such as ornithology (Digby et al. 2013), where passive acoustic methods are beginning to be recognized for their strengths and effectiveness to record rare species or species with low detectability.

## Supporting Information

**Appendix S1**. Species accumulation curve built by averaging Clench model parameters (*a* and *b*) of each species accumulation curve per site (grey points), exemplified for the forest gap. Dotted lines represent standard errors. **Table S1**. Time invested for the field management required to sample one forest plot. **Table S2**. Description of Clench equation parameters for single microhabitats in relation to temporal sampling patterns: *a* is the slope at the beginning of the sampling, *b* is a parameter related to the shape of the accumulation of new species during the sampling, *t* is the sampling effort, *a/b* equals the asymptotic species richness. **Table S3**. Description of Clench equation parameters for combinations of micro-habitats in relation to temporal sampling patterns: *a* is the slope at the beginning of the sampling, *b* is a parameter related to the shape of the accumulation of new species during the sampling, *t* is the sampling effort, *a/b* equals the asymptotic species richness. **Table S4**. Description of Clench equation parameters for the best sampling schemes found for km^2^ cells in relation to the number of plots: *a* is the slope at the beginning of the sampling, *b* is a parameter related to the shape of the accumulation of new species during the sampling, *t* is the sampling effort, *a/b* equals the asymptotic species richness.
