# Supplementary material for: Optimizing passive acoustic sampling of bats in forests
Source: Ecol Evol. 2014 Dec 2;4(24):4690–700. doi: 10.1002/ece3.1296 (PMC4278821; doi:10.1002/ece3.1296)
Supplement: Supplementary file 1 [file ece30004-4690-sd1.docx]

**SUPPORTING INFORMATION**

**Appendix S1**. Species accumulation curve built by averaging Clench model parameters (*a* and *b*) of each species accumulation curve per site (grey points), exemplified for the forest gap. Dotted lines represent standard errors.

**Table S1**. Time invested for the field management required to sample one forest plot.

GA: Forest gap, GR: Forest ground, CA: Canopy.

| **Sampling scheme** | **Driving time (min)** | **Walking time (min)** | **Detector installation (min)** | **Detector retrieval**  **(min)** | **Data download (min)** |
| --- | --- | --- | --- | --- | --- |
|  |  |  |  |  |  |
| GA + GR + CA Full night | 60 | 15 | 50 | 30 | 3.7 |
|  |  |  |  |  |  |
|  |  |  |  |  |  |
| GA + GR Full night | 60 | 15 | 10 | 10 | 2.2 |
|  |  |  |  |  |  |
|  |  |  |  |  |  |

**Table S2**. Description of Clench equation parameters for single micro-habitats in relation to temporal sampling patterns: *a* is the slope at the beginning of the sampling, *b* is a parameter related to the shape of the accumulation of new species during the sampling, *t* is the sampling effort, *a/b* equals the asymptotic species richness.

GA: Forest gap, GR: Forest ground, CA: Canopy.

| **Micro-habitat** | **Temporal sampling pattern** | **S(*t*) = *at*/(1+*bt*)** | | |
| --- | --- | --- | --- | --- |
|  |  | **a** | **b** | **a/b** |
| GA | Full night | 5.48 | 0.54 | 10.1 |
|  | 4 hours | 4.19 | 0.48 | 8.7 |
|  | 2-2 hours | 4.01 | 0.51 | 7.9 |
| GR | Full night | 4.29 | 0.51 | 8.4 |
|  | 4 hours | 3.23 | 0.42 | 7.7 |
|  | 2-2 hours | 2.95 | 0.41 | 7.2 |
| CA | Full night | 3.29 | 0.45 | 7.3 |
|  | 4 hours | 2.56 | 0.40 | 6.4 |
|  | 2-2 hours | 2.44 | 0.40 | 6.1 |

**Table S3**. Description of Clench equation parameters for combinations of micro-habitats in relation to temporal sampling patterns: *a* is the slope at the beginning of the sampling, *b* is a parameter related to the shape of the accumulation of new species during the sampling, *t* is the sampling effort, *a/b* equals the asymptotic species richness.

GA: Forest gap, GR: Forest ground, CA: Canopy.

| **Combination** | **Temporal sampling pattern** | **S(*t*) = *at*/(1+*bt*)** | | |
| --- | --- | --- | --- | --- |
|  |  | **a** | **b** | **a/b** |
| GA + GR + CA | Full night | 9.42 | 0.73 | 12.9 |
|  | 4 hours | 6.44 | 0.56 | 11.5 |
|  | 2-2 hours | 6.29 | 0.61 | 10.3 |
| GA + GR | Full night | 8.12 | 0.67 | 12.1 |
|  | 4 hours | 5.68 | 0.53 | 10.7 |
|  | 2-2 hours | 5.54 | 0.58 | 9.6 |
| GA + CA | Full night | 7.31 | 0.62 | 11.8 |
|  | 4 hours | 5.30 | 0.53 | 10.0 |
|  | 2-2 hours | 4.96 | 0.53 | 9.4 |
| GR + CA | Full night | 5.60 | 0.54 | 10.4 |
|  | 4 hours | 4.02 | 0.47 | 8.6 |
|  | 2-2 hours | 4.00 | 0.54 | 7.4 |

**Table S4**. Description of Clench equation parameters for the best sampling schemes found for km^2^ cells in relation to the number of plots: *a* is the slope at the beginning of the sampling, *b* is a parameter related to the shape of the accumulation of new species during the sampling, *t* is the sampling effort, *a/b* equals the asymptotic species richness.

GA: Forest gap, GR: Forest ground, CA: Canopy.

| **Sampling scheme** | **No. of plots** | **S(*t*) = *at*/(1+*bt*)** | | |
| --- | --- | --- | --- | --- |
|  |  | **a** | **b** | **a/b** |
| GA + GR + CA Full night | 1 | 9.42 | 0.73 | 12.90 |
|  | 2 | 16.19 | 1.16 | 13.96 |
|  | 3 | 22.70 | 1.59 | 14.28 |
|  | 4 | 29.65 | 2.06 | 14.39 |
| GA - GR Full night | 1 | 8.12 | 0.67 | 12.12 |
|  | 2 | 13.28 | 0.97 | 13.69 |
|  | 3 | 18.87 | 1.34 | 14.08 |
|  | 4 | 24.34 | 1.72 | 14.15 |
